# Psychometric evaluation of the Chinese version of new fear of the coronavirus questionnaire

**DOI:** 10.1097/MD.0000000000037282

**Published:** 2024-03-01

**Authors:** PeiJia Zha, Crystal Zhang, Rubab Qureshi, GuiGui Cai, LiHong Huang, Yang Liu

**Affiliations:** a Department of Nursing, School of Medicine, Xiamen University, Xiamen, China; b Division of Nursing Science, School of Nursing, Rutgers, the State University of New Jersey, Newark, NJ; c Princeton High School. Princeton, NJ.

**Keywords:** Chinese, COVID-19, new fear of the coronavirus questionnaire, reliability, validity

## Abstract

The fear of COVID-19 significantly impacting the health of people globally. This study translated newly developed measurement tool New Fear of the Coronavirus Questionnaire (New_FCQ) into Chinese language and evaluated the psychometric properties of the Chinese version of New_FCQ among Chinese population. A total of 522 participants were included in the study. Internal consistency, construct validity, criterion validity, and concurrent validity of the Chinese version of New_FCQ were assessed in this study. The Chinese version of New_FCQ had excellent internal consistency (α = 0.97) and exploratory factor analysis demonstrated one-dimensional structure of the Chinese version of New_FCQ. The preliminary criterion validity revealed statistically significant differences in the fear of COVID-19 scores based on age and education level (*P* = .002 and *P* = .03, respectively). The good concurrent validity also established with the Chinese version Fear of COVID-19 Scale(*P* < .001). Psychometric proportions of the Chinese version of New_FCQ were established, which exhibited sufficient validity and reliability among Chinese population.

## 1. Introduction

It has been more than 3 years since the first reported SARS-CoV-2 case also known as COVID-19 reached public awareness in central China. As of February 15, 2023, 756,135,075 confirmed cases of COVID-19 worldwide have been reported to World Health Organization, including 6,841,152 deaths.^[1]^ In China, from January 3, 2020 to November 16, 2023, there have been reported 772,011,164 confirmed cases of COVID-19 including 6,979,786 deaths.^[1]^ People’s physical health, mental health, and quality of life have all been impacted this pandemic in various ways.

Researchers from around the world have conducted numerous studies exploring the impact of COVID-19 on mental health. Most of the research employing quantitative measurements has primarily concentrated on examining the psychological stressors experienced by COVID-19 patients and front line healthcare workers, as well as the resulting outcomes for their mental well-being. These studies demonstrated that the COVID-19 patients have some psychological stressors, including fear, depression, anxiety, guilt, stigma, etc.^[2,3]^ Additionally, healthcare workers who were working on the front line have adverse psychological problems, including stress, anxiety, fear as well as fatigue caused by high-intensity work during the pandemic.^[4,5]^ Research indicated that stress, fear, and anxiety experienced by healthcare workers are not just immediate reactions to the pandemic’s challenges, but have evolved into long-term mental health issues.^[6]^ The persistent nature of COVID-19, coupled with the constant exposure of healthcare professionals to high-risk environments, has not only heightened their sense of the crisis but also placed them at a greater risk of long-term psychological effects.^[6]^ Understanding these impacts is important for developing strategies to support the mental health and resilience of those who are at the front line of battling this unprecedented global health challenge.

Central to these psychological responses is the emotion of fear.^[7]^ Fear is prevalent not only among patients grappling with the direct impacts of the virus but also pervasively affects healthcare providers. This shared experience of fear, bridging the gap between patients and healthcare providers, further amplifies the need for understanding and addressing these complex emotional responses within the context of the COVID-19 pandemic.

Fear is a negative normal emotional response of individuals to real or perceived threats, which is used to stimulate individuals to deal with potential threats or risks. It is an indispensable adaptive function in the process of survival and evolution.^[8]^ Fear of COVID-19 (FCV) mainly indicated having an infection or spreading the COVID-19 it to others.^[9]^ According to fear of COVID-19 studies, the more infected COVID-19 cases, the more fear people have, and the more likely they were suffering from poor sleep, anxiety, and other adverse conditions.^[10,11]^ FCV also associated to the severity of the COVID-19 incidences by the region, age, gender, and occupation.^[10–13]^

During the pandemic, the middle-aged people are less afraid of COVID-19 than the elderly.^[14,15]^ Regarding the gender, the fear level of women is higher than that of men,^[13]^ this difference is attribute to several factors, women might perceive themselves are more vulnerable to COVID-19 due various reasons, including societal roles, biological factors, or a combination of both. For instance, women often take on caregiving roles, both professionally in healthcare settings and personally within families, potentially increasing their exposure to the virus and concern for transmitting it to others.^[13,16]^ Additionally, women’s socialization may lead them to be more open about expressing fears and anxieties, whereas societal expectations often encourage men to suppress or underreport their fears.^[17]^ The study also found that healthcare workers are more afraid of COVID-19 than people in other occupations; front line pandemic prevention personnel generally feel nervous, anxious, and fearful in the process of fighting the epidemic. Insufficient training and shortage of protective equipment exposed healthcare workers to greater risk of COVID-19 and greater anxiety and fear.^[6,17,18]^ Overall, people have different degrees of fear in front of the pandemic. In general, socially vulnerable groups report a higher sensitivity to subjective assessments of fear, concern, and threats.^[10]^ These groups typically have less access to healthcare resources, security, and social support, which can amplify perceived threats and concerns. Without adequate resources or support systems, individuals in these groups may feel less equipped to handle or mitigate risks, leading to heightened fear and anxiety. Additionally, vulnerable populations are more likely to have experienced or be aware of instances of unfair treatment, bias, and injustice, either personally or within their community. This awareness can sensitize them to potential threats, making them more alert and cautious in their interactions and assessments of risk. The level of fear of COVID-19 in different countries is related to different levels of social background, cultural factors, and access to healthcare.^[19]^ Consequently, it is imperative for every country to devise approaches and establish measurement instruments tailored to their specific circumstances in order to effectively manage and mitigate the excessive fear associated with COVID-19.

In order to measure the fear of COVID-19, there were several research scientists developed and validated the measurement instruments during the pandemic. Since 2020, the measurement instruments have attracted attention in China. The most widely used instruments including fear of COVID-19 scale (FCV-19s),^[9]^ COVID stress scales (CSS),^[20]^ and fear of the coronavirus questionnaire (FCQ).^[21]^ More specifically, FCV-19s was developed to measure the perception of fear of COVID-19.^[9]^ CSS measures 5 factors (danger and contamination fears, fears about economic consequences, xenophobia, compulsive checking and reassurance seeking, and traumatic stress symptoms) and worries related to COVID-19.^[20]^ FCQ measures the 8 aspects about subjective worry, safety behaviors, and preferential attention of fear of COVID-19.^[21]^ Both FCV-19s and CSS have been translated into different languages (including Chinese) and applied in clinical practices.^[22–26]^

However, as fear can be elicited by a wide range of threats and generate defensive responses simultaneously, Mertens et al^[27]^ have noted fear could not just be pathological pathways, both socioeconomic and interpersonal components may operate as distinct or mediators of physiological manifestations of fear. According to Mertens et al,^[28]^ people are mostly concerned about the health of their loved ones and the economic consequences. People were to a lesser extent affected by mass hysteria, social consequences and spreading the virus unknowingly and losing their job. Additionally, less people were concerned about their own health. Therefore, it is crucial to include these components in the fear of COVID-19 measure instrument and to be able to keep the stability and accuracy of the instrument. The newly developed fear of the coronavirus questionnaire (New_FCQ)^[27]^ based on FCQ^[21]^ was to measure fear of health and fear of socio-economic consequences. The New_FCQ were compared with other 5 fear related instruments and demonstrated best fit of the data and captured all fear of COVID-19 factors.^[27]^ The original English version of FCQ consisted of one-dimensional structure and 8 items and demonstrated acceptable internal consistency (α = 0.77) in a sample of 439 Europe respondents. The New_FCQ consisted 16 items and 2-factors constructions, revealed good internal consistency (α = 0.85 and 0.80 respectively).

The English version of FCQ only has been applied to few research studies.^[27]^ To date, no studies have verified New_FCQ’s psychometric properties in China. The purpose of this study was to translate the New_FCQ instrument^[27]^ to the Chinese language and assess the reliability and validity in Chinese population. The hypotheses for this study as follow: firstly, the Chinese version of the New_FCQ will demonstrate a high level of reliability, indicating consistent results across various applications. Secondly, the instrument will exhibit strong validity, accurately reflecting the fear of COVID-19 among Chinese population. Results will add a linguistically congruent instrument to determine fare of COVID-19 based on client perceptions while giving voice to Chinese population, who are still experiencing the pandemic sufferings.

## 2. Method

### 2.1. Study design

This study was conducted in 2 phases. After obtaining the permission from the authors of the New_FCQ, the instrument was translated from English to Mandarin followed by the second phase psychometric testing of the Chinese version of New_FCQ. The second phase of the study was using a quantitative cross-sectional design and conducted the psychometric testing of the Chinese version of New_FCQ. As study occurred during COVID-19 pandemic lockdown, the online survey was distributed to study participants.

After approval by the Medical School of Xiamen University Institutional Review Board (XDYX2021027), we collected data through offline paper questionnaires and online questionnaires using a tool called “Wenjuanxing.”

### 2.2. Translation of the English version New_FCQ

Brislin translation-back-translation model^[29]^ was used to ensure accuracy of translation by using a bilingual college student to translated the original English version of New_FCQ to the Chinese language and using another bilingual high school student to translate blindly the Chinese version back to English. The translator is the Chinese citizen, and back translator is the American citizen, both of them born and raised in China and familiar with how Chinese and English languages are used in contemporary Chinese society.^[30]^ They have worked in various social study research project and also familiar with Chinese language, culture and society.

### 2.3. Content and face validity assessment

Content validity was assessed on the Chinese version New_FCQ by 5 experts including one epidemiologist and 4 nursing scientists. The 5 experts were invited to further examine the Chinese version New_FCQ on reasonability, suitability, attractiveness, and conciseness as well as comprehensiveness.^[30]^ The item level content validity index (I-CVI) of the instrument was computed for each item on the Chinese version New_FCQ. The scores for I-CVI suggested I-CVI of .78 or higher with 3 or more experts could be considered as a good content validity.^[31]^ The I-CVI of the item ranged from 0.79 to 1 for the Chinese version New_FCQ, which demonstrated all items had a favorable content validity.

In a preliminary assessment of the face validity, the translated version was pilot test to thirty Chinese to confirm the readability and feasibility, thirty participants were instructed to comment on the appropriateness, clarity of language and other observations regarding any items on the instrument through an open-ended question at the end of the survey. According comments of 30 participants provided, the Chinese version of New_FCQ were appropriately adjusted and modified to form the final Chinese version of New_FCQ.

### 2.4. Sample and recruitment

Convenience sampling was used to collect a study sample of 552 participants. The sample size was estimated based on probability proportionate, including expected prevalence, margin of error, confidence level, and standard deviation.^[32]^ Thus, the minimum sample size was 461, taking into account the 20% attrition rate. A total of 552 participants were included in the sample to construct the final analyses.

Participant inclusion criteria were: 18 years and over, Chinese national residing in the China, can read and write in Chinese, experienced COVID-19 pandemic, and have internet access and device to answer an online survey. Exclusion criteria included a diagnosis of any mental health disorder or critical illness. Study participants were recruited through WeChat and QQ (social media platform). All recruited study participants provided electronic consent, which explaining the purpose and voluntary nature of the study. Incomplete and subsequently submitted surveys with the same account number were excluded from the analysis.

### 2.5. Demographic questionnaire

The demographic questionnaire included thirteen items about participants’ age, gender, marital status, level of education, risk level of COVID-19 in the current location, employment status, minor below 18, daily time spend to pay attention on COVID-19, knowledge about COVID-19, any experience of infection with COVID-19, family members infected by COVID-19, experience of quarantine and hospitalized caused by COVID-19. The demographic questions were aimed to assess the criterion relationships between demographic factors and the Chinese version of New_FCQ.^[33]^

### 2.6. New fear of COVID-19 questionnaire (New_FCQ)

In this study, the 16-item New_FCQ was adopted and the English version of was translated into the Chinese version after obtaining permission from the authors of the New_FCQ The Chinese version of New_FCQ also consists of sixteen items, each item is rated on a 5-point Likert type scale from 1 (strongly disagree) to 5 (strongly agree). Following with the English Version, the Chinese version of New_FCQ composites sum of item scores on 2 factor constructs: fear of health (item 1, 2, 5, 8, 9, 10, and 12) and fear of socio-economic consequences (item 3, 4, 6, 7, 11, 13, 14, 15, and 16), which higher sum scores indicating greater level of fear of health and fear of socio-economic consequences.

### 2.7. Fear of COVID-19 scale (FCV-19s)

The Chinese version of FCV-19s used in study to examine the concurrent validity with the Chinese version of New_FCQ. Developed by Ahorsu et al,^[9]^ the FCV-19s consists 7 items to measure the individuals’ perception of fear of COVID-19. All items are rated on 5-point Liker scale from “strongly disagree” to “strongly agree.” The higher composite score indicating the greater level of fear of COVID-19. The Chinese version of FCV-19 demonstrating the good internal consistency reliability (α = 0.92) and composite reliability (CR = 0.92).^[24]^

### 2.8. Data analysis

Data were analyzed via R 4.2.2. Descriptive statistics were applied to summarize participants’ demographic characteristics. Pearson correlation, independent t-test and one way analysis of variance (ANOVA) tests were performed to assess the mean differences on the Chinese version of New_FCQ scores among study participants across different social demographic groups.

The internal consistency reliability of the Chinese version of New_FCQ was assessed by Cronbach alpha coefficient and item-to-total correlation coefficient (ITC) and for homogeneity. ITC value >0.3 is the minimum acceptable value for items in a measurement instrument, and Cronbach alpha coefficient values >0.7 are considered satisfactory.^[34]^ In order to maximize the reliability of the Chinese version of New_FCQ, the threshold for the ITC was set at 0.5 for this study, only the items with an ITC >0.5 were retained for further analyses.

Construct validity was examined by exploratory factor analysis (EFA). For the Chinese version of New_FCQ, the laten structure of the items and preliminary indications of a theoretical structural solution were examined using the EFA.^[35,36]^ Prior to completing confirmatory factor analysis, the sampling adequacy of the data and sufficient sample size were assessed by the Kaiser–Meyer–Olkin (KMO) value.^[34]^ Additionally, the homogeneity of variances of the data was assessed by the Bartlett test of sphericity in this study.^[34]^

The criteria used to determine the number of factors for each construct of the instrument were eigenvalues larger than 1 and the proportion of total variance accounted for by the factors.^[34]^ A maximum likelihood method was used to estimate the factor loadings, items with loadings of 0.4 or higher were retained as factors members.^[34]^

In addition, the Chinese version New_FCQ validity testing was also based on assessing the criterion relationships^[33]^ between demographic factors and scores in the instrument using generalized linear model with hypothesis that demographic information of study participants would affect their fear of COVID-19. The rationale for the choice of sociodemographic factors as based on empirical evidence of that COVID-19 is a global health crisis that impact various of countries, it is crucial to have a cross- national understanding of potential sociodemographic factors and fear of COVID-19.^[12]^ For this purpose, the sociodemographic factors, which showed the significant differences on the Chinese version of New_FCQ scores, was extracted for criterion validity analyzes. Concurrent validity was assessed by Pearson correlation with FCVs-19. Correlation coefficient under 0.3 were considered to be weak, between 0.3 and 0.5 were considered as moderate, and those >0.5 were consider strong.^[37]^

## 3. Results

### 3.1. Characteristics of study participants

Data collection of this study occurred from November 2021 to May 2022. A total of 552 sample data were received, after excluding surveys with incomplete answers, a total of 522 were included for analysis. Table 1 shows the participants’ demographic information. Participants’ age ranged from 18 to 55 years with an average of 26.57 (SD = 6.73). Participants gender were about evenly split between male (49.43%, *n* = 258) and female (50.57%, *n* = 264). More than half of the participants were single (58.62%, *n* = 306) and had college education (54.02%, *n* = 282). About 34% of participants were students (*n* = 176). Majority of participants reported they lived in low-risk COVID-19 locations (78.74%, *n* = 411) and don’t have child (59.00%, *n* = 308). Abou 38% (*n* = 193) of participants reported they were spending between half and 1 hour daily to pay attention on COVID-19 related information, and more than half (58.05%, *n* = 303) of them having basic knowledge about COVID-19. Majority of the participants reported they never infected by COVID-19 (99.22%, *n* = 519), they don’t have any family member infected by COVID-19 (99.43%, *n* = 519), they never quantized caused by COVID-19 (89.27%, *n* = 466) and they never hospitalized caused by COVID-19 (99.23%, *n* = 518).

**Table 1:** Demographic characteristics of study participants (*n* = 522).

Demographic characteristics	*n* (%)	*P* value (Chinese version of New_FCQ)
Age (mean SD)	26.57 (6.73)	0.002
Gender		
Male	258 (49.43)	0.82
Female	264 (50.57)	
Marital status		
Married	210 (40.23)	0.18
Single	306 (58.62)	
Other	6 (1.15)	
Education		
Below High and High School	99 (18.97)	0.03
2-year college	113 (21.65)	
4-year college	282 (54.02)	
Above College	28 (5.36)	
Risk level of COVID-19 in the current location		
High	54 (10.34)	0.22
Medium	57 (10.92)	
Low	411 (78.74)	
Employment status		
Employed	316 (60.54)	0.44
Unemployed	30 (5.75)	
Student	176 (33.72)	
Minor under 18		
Yes	308 (59.00)	0.30
No	214 (41.00)	
Daily time spend to pay attention on COVID-19		
<0.5 h	185 (35.44)	0.09
0.5–1 h	193 (36.97)	
1–2 h	94 (18.01)	
>2 h	50 (9.58)	
Knowledge about COVID-19		
Adequate knowledge	127 (24.33)	0.29
Basic knowledge	303 (58.05)	
Minimal knowledge	85 (16.28)	
No knowledge	7 (1.34)	
Infection with COVID-19		
Yes	4 (0.77)	0.06
No	518 (99.23)	
Family members infected by COVID-19		
Yes	3 (0.57)	1.62
No	519 (99.43)	
Quarantine caused by COVID-19		
Yes	56 (10.73)	0.07
No	466 (89.27)	
Hospitalized caused by COVID-19		
Yes	4 (0.77)	0.06
No	518 (99.23)	

Percentages may not sum to 100 due to missing data and rounding

New_FCQ = new fear of the coronavirus questionnaire

According to Pearson correlation test and ANOVA test, there were significant negative correlation between the Chinese version of New_FCQ scores and age (*r* = −0.13, *P* = .002), suggesting that age increase as the fear of COVID-19 decrease. ANOVA results showed a significant difference of the Chinese version of New_FCQ scores on education level (*F* (3, 512) = 2.94, *P* = .03). Post hoc comparison using the Sidak demonstrated that the Chinese version of New_FCQ score for below high school education level participants (*M* = 57.34; SD = 18.81) were significantly different than 2-year college education level (*M* = 50.80, SD = 19.35, *P* = .03). However, the Chinese version of New_FCQ scores did not significantly differ from other education level. Additionally, there were no significant difference of the Chinese version of New_FCQ scores was found among other demographic characteristics (Table 1).

### 3.2. Internal consistency reliability

The mean for each individual item of the Chinese Version of New_FCQ ranged from 3.15 to 3.60. Cronbach alphas for the Chinese Version of New_FCQ reached 0.97, which demonstrating excellent internal consistency. The item-to-total correlation of Chinese Version of New_FCQ ranged from 0.70 to 0.87, demonstrating good interrelatedness of all the items (See Table 2 for details). Additionally, fear of health and fear of socio-economic consequences subscales revealed the Cronbach alpha coefficients of 0.94 and 0.95 respectively. Thus, total 16 items of the Chinese version of New_FCQ were retained for the rest of the data analyses.

**Table 2:** Item analysis and factor structure for the Chinese version of New_FCQ (*n* = 522).

Item	Item-total correlation	Alpha without item
1. I am worried about vulnerable loved ones (e.g., parents, grandparents) becoming infected by the coronavirus.	0.79	0.969
2. I am worried that the healthcare system will be overloaded because of COVID-19.	0.82	0.968
3. I am worried that the economy will collapse because of COVID-19.	0.84	0.968
4. I am worried that COVID-19 is causing mass panic.	0.85	0.968
5. I am worried about getting infected and sick due to COVID-19.	0.84	0.968
6. I am worried that society will break down because of COVID-19.	0.87	0.968
7. I am worried about losing my job/not finding a job because of COVID-19.	0.78	0.969
8. I am worried that the coronavirus will mutate into a deadlier strain or never disappearing from the population.	0.82	0.968
9. I am worried about unknowingly spreading the coronavirus.	0.85	0.968
10. I am worried that others will not continue to follow the rules.	0.78	0.969
11. I am worried about being in quarantine or lockdown for a long time.	0.82	0.968
12. I am worried that the government or health authorities will not act/are not acting responsibly.	0.77	0.969
13. I am worried that there will be shortages of food or other supplies.	0.83	0.968
14. I am worried that I cannot pick up my normal routines again (e.g., going to school, work, sports).	0.81	0.968
15. I am worried not being able to travel due to COVID-19.	0.70	0.970
16. I am worried that fake or inaccurate news is being spread about COVID-19.	0.79	0.969

New_FCQ = new fear of the coronavirus questionnaire

### 3.3. Construct validity

**Figure 1.**
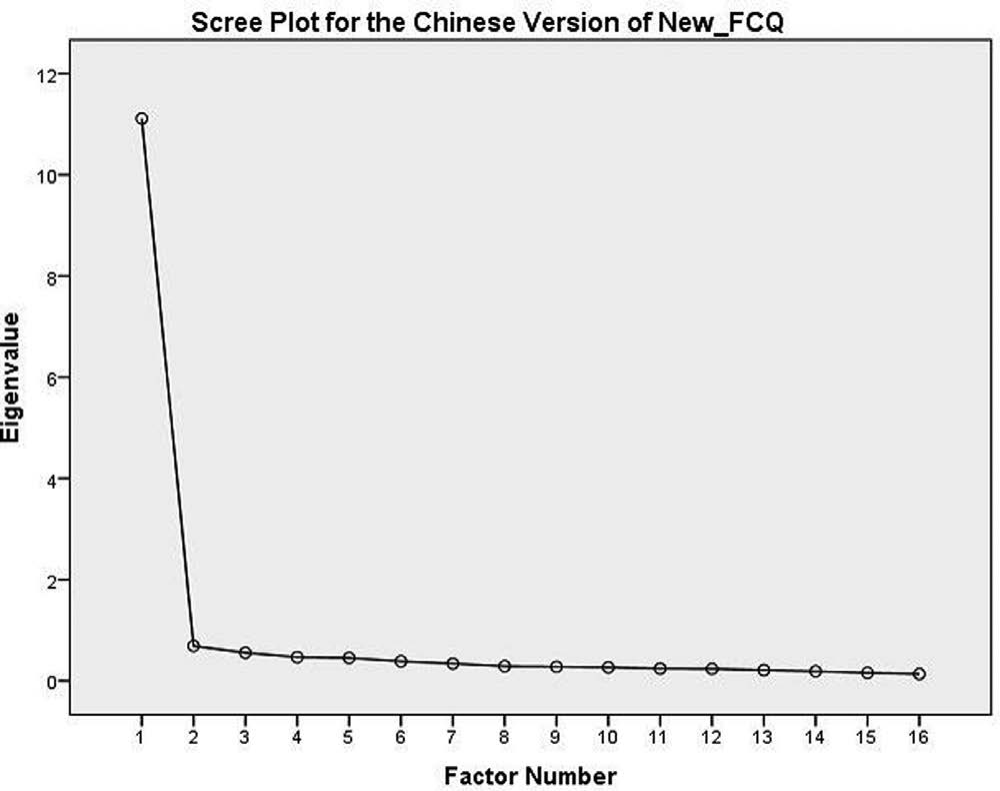
Displays a scree plot used in exploratory factor analysis, used to determine the optimal number of factors for the analysis. This graphical representation demonstrates the eigenvalues of each of the 16 items from the Chinese version of New_FCQ. In the plot, eigenvalues are arranged in descending order from left to right. A major observation is the gradual tapering off of the plot line. Initially, a significant drop in eigenvalues is noticeable between the first and second components. However, from the second component onwards, there is a minimal rate of change or slope across the remaining components, extending to the sixteenth. The point where changes in eigenvalues become less pronounced is indicative of the number of components to retain. In this case, the sharp decrease from the first to the second component, followed by a leveling off, suggests that retaining 1 component would be most appropriate for the Chinese version of the New_FCQ.

The KMO test results demonstrated adequate sampling (KMO = 0.97).^[34]^ According to Bartlett test of sphericity, the equality in variances were assuring (χ²=8207.51, *P* < .001). Unlike the English version of New_FCQ, the construct validity using maximum likelihood factoring analysis only supported the one-dimensional structure of the Chinese version of New_FCQ. This one-dimensional structure explained 64.45% of the cumulative contribution rate with eigenvalue of 11.11. Following Varimax rotation, all factors were having factor loading >0.4 (See Table 3 for details). Inspection of the scree plot (Fig. 1) also supported the unidimensional structure of the Chinese version of New_FCQ. Therefore, the one-factor model as the best model for the Chinese version of New_FCQ.

**Table 3:** Factor analysis of the Chinese version of New_FCQ (*n* = 522).

Item	Factor matrix	Communality
1. I am worried about vulnerable loved ones (e.g., parents, grandparents) becoming infected by the coronavirus	0.80	0.65
2. I am worried that the healthcare system will be overloaded because of COVID-19	0.83	0.70
3. I am worried that the economy will collapse because of COVID-19	0.86	0.73
4. I am worried that COVID-19 is causing mass panic	0.87	0.76
5. I am worried about getting infected and sick due to COVID-19	0.85	0.72
6. I am worried that society will break down because of COVID-19	0.88	0.78
7. I am worried about losing my job/not finding a job because of COVID-19	0.80	0.64
8. I am worried that the coronavirus will mutate into a deadlier strain or never disappearing from the population	0.83	0.68
9. I am worried about unknowingly spreading the coronavirus	0.86	0.73
10. I am worried that others will not continue to follow the rules	0.79	0.62
11. I am worried about being in quarantine or lockdown for a long time	0.83	0.68
12. I am worried that the government or health authorities will not act/are not acting responsibly	0.77	0.60
13. I am worried that there will be shortages of food or other supplies	0.84	0.70
14. I am worried that I cannot pick up my normal routines again (e.g., going to school, work, sports)	0.82	0.67
15. I am worried not being able to travel due to COVID-19	0.71	0.50
16. I am worried that fake or inaccurate news is being spread about COVID-19	0.80	0.63

New_FCQ = new fear of the coronavirus questionnaire

### 3.4. Criterion validity

Based on series bivariate analyses, gender, marital status, and other related sociodemographic factors did not reveal the significant association with the Chinese version of New_FCQ. Only the age and level of education revealed the significant association with the Chinese version of New_FCQ scores. The generalized linear model showed that age was negatively significant associated with the Chinese version of New_FCQ scores (*P* = .002). One year age increasing was associated with 0.34 point of decrease of the Chinese version of New_FCQ. Additionally, the Chinese version of New_FCQ scores was affected by level of education of study participants (*P* = .03). About 1 unit of level of education decreasing was associated with 8.80 points increase of the Chinese version of New_FCQ. However, there were no significant differences found between different level of education. These results provide preliminary evidence for the relationship between sociodemographic factors impacts on the fear of COVID-19.

### 3.5. Concurrent validity

The Pearson correlation coefficient demonstrated the 16-item Chinese version of New_FCQ had good concurrent validity with the Chinese version FCV-19s (*R* = 0.73, *P* < .001).

## 4. Discussion

The objectives of this study were to translate and explore the psychometric properties of the Chinese version of New_FCQ among Chinese population. The findings of this study demonstrated that the Chinese version of New_FCQ having acceptable psychometric properties for measuring the fear of COVID-19 among Chinese population.

The Chinese version of New_FCQ is a 16-item self-reported instrument. Content validity was verified by experts in this study. The overall Cronbach alpha coefficient (0.97) of the Chinese version New_FCQ was higher than the original English version.^[27]^ Item-total correlations, serve as a pertinent indicator for assessing the suitability of scales across diverse populations.^[38]^ In the mentioned study, item-total correlations were examined to ensure compliance with quality standards. Consequently, the Chinese version of the New_FCQ exhibits both internal consistency and mutual exclusivity.

To our knowledge, this is the first study that translated and assessed the construct validity of the New_FCQ among Chinese population. In this study, all 16 items met the loading criteria and loaded significantly on one factor in EFA. The internal structure of the Chinese version of NEW_FCQ is clearly one-dimensional, measuring the fear of COVID-19. The difference in the factor loading was observed between the English and the Chinese version of New_FCQ. Acknowledge the excellent internal consistency of the Chinese version of New_FCQ, as well as the KMO value and spherical degree, the reason for this discrepancy might be high correlation between the items (between 0.51 and 0.81) and large volume of shared variances in terms of dimensional structure.

Additionally, the one-factor structure finding was fitting the data significantly for this sample. These 16 items were related to each other and their dimension was unique. As long as the COVID-19 is active, fear is a proper feeling. However, it is crucial that fear of COVID-19 and its associated safety behaviors decrease once the situation is under control. Otherwise, chronic fear could lead to undesirable outcomes like psychological distress at the individual level and an economic recession at societal level.^[28]^ Additionally, the cultural or contextual differences between the original population and the Chinese population influenced the results. Different cultural backgrounds or contextual factors can impact how individuals interpret and respond to the items, leading to variations in the underlying structure of the instrument. More appropriate structural models were needs to close the gap between the cultural adaptation and rigor of different version of a measurement instrument.^[39]^

There were few measurement instruments to measure the fear of COVID-19, the Chinese version FCV-19s is commonly used in China, this instrument was used to examine concurrent validity of the Chinese version of FCQ. The Pearson correlation coefficient between the Chinese version FCV-19s and the Chinese version of New_FCQ was 0.73 (*P* < .001) and reached the quality criteria, which demonstrated that the 2 scales showed good consistency.

A significant association between age, level of education, and fear of COVID-19 were found in this study. The study results suggested an inverse association between age and COVID-19 fear, which was contrary with previous studies.^[14,15]^ The age acknowledged in this study was ranged from 18 to 55. In these specific age range, majority of them played multiple roles in their family and the society. Their lives are filled with multiple functions, they therefore have more stress. This may explain why people in this age group have less fear of COVID-19 than those in other age range. Additionally, resilience and emotional stability associated with older age, their life experiences and coping mechanisms developed over time can influence one’s response to pandemics,^[40]^ aligning with this study findings that older individuals exhibited lower level of fear. Additionally, the study results also suggested inverse association between level of education and fear of COVID-19. This result was consistent with previous research.^[14]^ People with a higher level of education are more knowledge about COVID-19 and COVID-19 related preventions. They paid more attention and more accessible to COVID-19 related information, and can better discern the false information. Therefore, the people having higher level of education will have lower level of COVID-19 fear. Future studies should examine the how the different aspects of fear of COVID-19 are related to relevant health outcomes, especially the mental health.^[27]^

This result confirmed the social and cultural differentially constituted within the contexts of people’s lives between the original and the Chinese versions of the instrument. Another rationale for this discrepancy is the location. The COVID-19 has had a greater impact in some countries than others. For example, there was a significant disparity in the volume of cases between the European Union and United States from March 2020 to March 2021.^[41]^ During the same time, China remained locked down and quarantine for new infected cases, the incidence of COVID-19 was very low when compared with European Union and United States. This might result into different levels of threat perception and fear in these regions.^[28]^

Interestingly, in this study, gender and other sociodemographic factors did not reveal a significant difference in the fear of COVID-19 among the Chinese population. This could be attributed to the societal and cultural norms prevalent in China, where collective resilience and responses to crises may overshadow individual differences typically observed in other cultures.^[42]^ In Chinese society, there is often a strong emphasis on collective well-being and unified response to national challenges, which might reduce the prominence of gender-based differences and other sociodemographic-based differences in the perception of fear. Additionally, the uniform public health measures and government control across the China may have contributed to a more homogenized perception of the pandemic’s threat among Chinese population.

### 4.1. Limitations

The main limitation of this study was the data collection occurred during lockdown time and people remained low-risk during the time, most of participants complete the questionnaire at home, the fear of COVID-19 might be varied. In addition, due to greater homogeneity in the sample did not being infected by COVID-19, the perceptions of fear of COVID-19 also might be varied. The fear of COVID-19 was significant associated with intensity of the COVID-19 cases.^[43]^ The final limitation is that the instrument was designed for the general public in Europe. It is difficult to draw conclusions about the acceptance of the instrument based on one open-ended question at the end of the survey. Despite these limitations, this study provided preliminary validity and reliability evidence for the Chinese version of New_FCQ. At this point, the Chines version of New_FCQ offers straightforward and inexpensive instrument that could easily permit mass testing in significant population-based epidemiological studies, also enabling for cross-cultural comparisons.

## 5. Conclusion

The findings of study show that the Chinese version of New_FCQ can be used to measure the fear of COVID-19. The Chines version of New_FCQ had excellent internal consistency, good concurrent validity, and established criterion validity in Chinese population. More studies to further assess the rigor of structure of the Chinese version of New_FCQ should be considered for future research. The utilization of the new scale in research and practice has the potential to shed light on the Chinese population’s fears surrounding COVID-19, thereby aiding in efforts to mitigate such fear. Furthermore, its application can serve as a foundational basis for future COVID-19 management and intervention strategies.

## Author Contributions

**Methodology:** PeiJia Zha, Yang Liu.

**Writing—original draft:** PeiJia Zha, Crystal Zhang.

**Writing—review and editing:** PeiJia Zha, Rubab Qureshi, LiHong Huang, Yang Liu.

**Conceptualization:** GuiGui Cai, Yang Liu.

**Investigation:** GuiGui Cai, Yang Liu.

**Formal analysis:** Yang Liu.

**Funding acquisition:** Yang Liu.

**Supervision:** Yang Liu.
